# Study protocol: The Intensive Care Outcome Network ('ICON') study

**DOI:** 10.1186/1472-6963-8-132

**Published:** 2008-06-17

**Authors:** John A Griffiths, Kayleigh Morgan, Vicki S Barber, J Duncan Young

**Affiliations:** 1 ICS Trials Group, Kadoorie Centre, John Radcliffe Hospital, Headley Way, Oxford OX3 9DU, UK

## Abstract

**Background:**

Extended follow-up of survivors of ICU treatment has shown many patients suffer long-term physical and psychological consequences that affect their health-related quality of life. The current lack of rigorous longitudinal studies means that the true prevalence of these physical and psychological problems remains undetermined.

**Methods/Design:**

The ICON (Intensive Care Outcome Network) study is a multi-centre, longitudinal study of survivors of critical illness. Patients will be recruited prior to hospital discharge from 20–30 ICUs in the UK and will be assessed at 3, 6, and 12 months following ICU discharge for health-related quality of life as measured by the Short Form-36 (SF-36) and the EuroQoL (EQ-5D); anxiety and depression as measured by the Hospital Anxiety and Depression Scale (HADS); and post traumatic stress disorder (PTSD) symptoms as measured by the PTSD Civilian Checklist (PCL-C). Postal questionnaires will be used.

**Discussion:**

The ICON study will create a valuable UK database detailing the prevalence of physical and psychological morbidity experienced by patients as they recover from critical illness. Knowledge of the prevalence of physical and psychological morbidity in ICU survivors is important because research to generate models of causality, prognosis and treatment effects is dependent on accurate determination of prevalence. The results will also inform economic modelling of the long-term burden of critical illness.

**Trial Registration:**

ISRCTN69112866

## Background

Research on the effectiveness of intensive care treatment has traditionally focused on mortality [1-3]. The cumulative 12 month mortality of survivors of ICU treatment in the UK varies between 35% and 43% [4,5]. Over the five years after an ICU admission these patients also have an excess risk of death when compared to an age and sex matched population [6]

However, apart from this excess mortality, recent research has confirmed that survivors of ICU treatment continue to experience both physical [7], and psychological problems [4,8,9] for some time after discharge from ICU [7,10]. The reported prevalence of anxiety and depressive problems in ICU survivors ranges from 12% to 43% (for anxiety) [4,11], and 10% to 30% (for depression) [4,11]. Two recent reviews estimate that 5% to 64% of intensive care patients may develop either post traumatic stress disorder (PTSD) or its associated symptoms that may endure for a number of years [12,13]. However, the studies reporting on the rates of PTSD and PTSD symptoms in ICU survivors vary considerably in their case mix, demographic variables, and method and timing of PTSD assessment [12,13]. All these factors complicate comparisons between studies and limit quantitative synthesis of their findings. As a result, the extent to which the consequences of critical illness or treatments received in the ICU contribute to the prevalence of PTSD symptoms during recovery is currently poorly understood. Thus, well-designed studies are needed to determine accurate prevalence rates of PTSD in survivors of ICU treatment.

The extended physical and psychological morbidity seen after critical illness has the potential to affect a patient's quality of life. Quality of life is determined by several factors including health status, social relationships, employment status and the well being of others. Health status measurements assess physical, physiologic and psychological states or function, and where these measurements overlap with components of quality of life is defined as 'Health-Related Quality of Life' (HRQoL) [14]. Studies assessing HRQoL after intensive care suggest that this improves over time [15,16], but is worse than before admission to ICU [16], and worse than general population norms [4,15,17]. However, the current literature assessing HRQoL among ICU survivors [1,3,14,18] has limitations. First existing UK studies tend to be small and from single centres. The two largest studies collected data on only 173 patients 12 months post-discharge [19] and 143 patients 3 months post-discharge [4]. Both these studies were conducted in single centres. Second, the existing studies reporting on HRQoL in ICU survivors have employed a wide number of different outcome measures, making synthesis of the results impossible. Third, the majority of the studies lack sufficient rigour. Heyland et al evaluated 64 studies assessing HRQoL in ICU survivors (January 1992 – July 1995). Only three studies (5%) met their pre-set methodological standards [14]. A major shortcoming was the lack of validation of the HRQoL measures. Hayes et al reached a similar conclusion in their systematic review [1]. The SF-36 and EQ-5D appear to be the strongest of the existing generic measures because they have been validated extensively in other patient populations and are straightforward to administer [1,3,18]. Both the SF-36 and the EQ-5D also have the advantage that utilities can be obtained which can be used to calculate quality-adjusted life years in conjunction with survival data [3,19,20]. Despite these recommendations the SF-36 and EQ-5D measures have yet to be used (or extensively validated) in a large, representative sample of survivors of adult, general intensive care in the UK [1,3]. Therefore, a UK multicentre study applying these outcome measures to assess longer-term HRQoL in survivors of ICU treatment is long overdue.

### Aims and objectives of the study

The ICON study aims to:

Define the longer-term outcomes of a large, representative sample of survivors of adult, general ICU care in the UK quantitatively in terms of their survival, HRQoL and psychological morbidity 3, 6 and 12 months after ICU discharge.

Compare the mortality of survivors of adult, general ICU care with age-, sex- and condition-matched populations of people who have not experienced ICU care.

## Methods/Design

This is a multi-centre, longitudinal study.

### Ethics Approval

Ethics approval was granted from Oxfordshire Research Ethics Committee B (Ref:06/Q1605/17).

### Participants

Participants meeting the inclusion criteria will be recruited from 20–30 intensive care units in the UK.

### Inclusion criteria

To be eligible for enrolment in the ICON study, participants must have experienced at least 24 hours of level three dependency care (ICU) at any time during their hospital stay and who survive until the time of hospital discharge.

### Exclusion criteria

Patients will be excluded if they are: aged under 16 years; unable to complete questionnaires; foreign nationals; in residential care; unable or unwilling to consent; or if their life status cannot be traced (i.e. have no GP or NHS numbers).

### Outcome assessments

Outcomes assessed in the ICON study include mortality, functional status, HRQoL and psychological outcomes. Relevant outcomes are assessed at 3, 12 and 24 months after ICU discharge (Table 1).

**Table 1:** Outcomes assessed at each follow-up point in the Intensive Care Outcome Network (ICON) study

**Outcome**	**Instrument(s)**	**Time required **^a ^(minutes)
Health-related quality of life	SF-36	12
	EQ-5D	2
Anxiety and Depression	HADS survey	6
PTSD symptoms	PCL-C	5

SF-36, Medical Outcomes Study Short Form 36-Item Health Survey; EQ-5D, EuroQOL instrument; HADS, Hospital Anxiety and Depression Scale; PCL-C, PTSD Civilian Checklist

^a ^Derived from pilot testing

Outcomes are assessed with standardised instruments, most of which are used widely in critical care research. HRQoL will be measured by the SF-36 [21,24] and the EQ-5D [22]. The SF-36 is a comprehensive, generic 36-item questionnaire [26,27] that appears to be an acceptable, reliable and valid tool for use in the ICU population and its use for quality of life assessment following critical illness has been recommended [23,15]. The SF-36 contains 36 items to measure 8 domains: physical functioning, role limitations due to physical problems, bodily pain, general health perceptions, energy/vitality, social functioning, role limitations due to emotional problems, and mental health [21].

The EQ-5D (EuroQoL) has been proved to be a useful tool in a mixed critical care population [22]. The EQ-5D comprises two parts: the EQ-5D self-classifier and the EQ-VAS. The self-classifier is self-reported description of health problems according to a 5 dimensional classification i.e. mobility, self-care, usual activities, pain/discomfort and anxiety/depression. The EQ-VAS is a self-rated health status using a visual analogue scale (VAS). The EQ-VAS is similar to a thermometer and records perceptions of participants own current overall health. The VAS scale is graduated from 0 (the worst imaginable health state) to 100 (the best imaginable state) [24]. Two EQ-VAS will be included in the three month questionnaire pack. The first scale one will ask patients to rate their health immediately prior to their hospital admission that resulted in the ICU admission. The second scale will be used to rate their health current health status.

Anxiety and depression will be assessed by the HADS (Hospital Anxiety and Depression Scale) [25]. The HADS has been validated in the critical care population [4,9,11,26]. The HADS questionnaire contains 14 statements and scoring results in scales of 0–21 for anxiety and depression respectively. Scores of 8–10 indicate the possibility of anxiety or depression, and 11 and above indicate that these are likely to be present.

PTSD should ideally be diagnosed using consistent criteria and reliable instruments that exhibit high inter-rater reliability, are stable over time and are able to assess individual patients presenting with wide symptom variance [12]. Currently, the PTSD diagnostic gold standard remains a structured clinical interview to predefined criteria (e.g. DSM-IV). For obvious reasons, standardised interview is not always practically possible at ICU follow-up, and the administration of self-report inventories allows the identification of PTSD symptomatology. The ICON study will assess PTSD symptomatology using the PTSD checklist (civilian) (PCL-C). The PCL-C is a 17-item self-report measure of the 17 DSM-IV symptoms of PTSD assessing all core symptoms of PTSD i.e. intrusion, avoidance and hyperarousal. It takes minutes to complete and is a reliable and validated tool with excellent psychometric properties. A correlation of 0.93 between the total PCL score and structured interview with the Clinician-Administered PTSD Scale (CAPS) has been demonstrated (Blanchard et al), with a diagnostic efficiency of 0.9 versus the CAPS. A score of 45 or greater on the PCL-C has been recommended as a cut off point for high PTSD symptom load. A total PCL-C cut-off score of 50 has a sensitivity of 0.6 and a specificity of 0.99 of diagnosing PTSD when compared to Structured Clinical Interview for DSM-IV [28,29].

In the event of a patient scoring 45 or greater on the PCL-C and or 11–14 (moderate risk) or 15–21 (severe risk) on either aspect of the HADS, a standard letter will be sent to the patient's GP to highlight that they have demonstrated significant risk of PTSD and or anxiety or depression on the relevant screening tools. However, the exact inventory scores will not be given to the GP. If the patient did not consent for a warning letter to be sent to the GP in the event of a high inventory score, a standard letter will be sent to the patient instead. This letter will inform the patient that their questionnaire results were inconclusive and they are recommended to visit their GP to discuss their psychological health.

### Procedure

Lists of patients meeting the study inclusion criteria will be sent from the participating hospitals to the trial office, which then submits this list to the Office of National Statistics (ONS). The ONS then performs two checks on the data. The first check cleans the list to maximise the identification of individual patients on the ONS databases; the second check identifies the patients that have died since hospital discharge. From the list of surviving patients generated by the ONS, contact with the patient's GP is made to act as a further check to confirm that the patient is still alive. Adopting this approach will hopefully prevent a questionnaire pack being mailed to a deceased patient and unnecessary distress being caused to their relative.

The three-month mailing pack consists of an introductory letter to the study, a patient information sheet, consent form, questionnaire set and a FREEPOST envelope. If the questionnaire pack is not returned, a reminder mailing is posted four weeks later. Once a patient is enrolled into the study, they will receive further study packs at 12 and 24 months post ICU discharge as long as they are not subsequently identified as deceased by the pre-mailing ONS check. The 12 and 24 month mailing packs comprise a reminder letter of the study, the relevant questionnaire packs and a FREEPOST envelope.

Again, if the patient has not returned either pack 4 weeks after the initial mailing, a second pack will be sent. If the questionnaire packs are not returned after the second mailing, no further attempts will be made to contact the patient.

### Trial recruitment and baseline measurements

All patients discharged from all participating units will be given a generic discharge letter introducing the study to them and the possibility that they may receive a mailing from the ICON study team in the coming months. Data will be collected on patient demographics, ICU diagnosis, ICU stay, severity of illness using the Acute Physiology, and Chronic Health Evaluation (APACHE II) system [30] and APACHE II co-morbidity score.

### Sample size

The minimum target total survivor recruitment for the study would be at least 1000 survivors per year in the first year returning all questionnaires. With this sample size, the ICON study would be the largest existing prospective cohort study of long-term outcomes in ICU survivors.

### Data collection

Participating ICUs will submit monthly lists of patient names, hospital numbers, CMP numbers, dates of birth, addresses, and both hospital/ICU admission and discharge dates to the trial office. Just prior to a patient reaching the 3 months post-ICU discharge date, the trial office will contact the patient's GP to check the patient status on the relevant patient administration system and ensure eligibility for trial entry. Once it is confirmed that the patient was successfully discharged from hospital and is still alive, a three month study pack is mailed. ICNARC (Intensive Care National Research and Education Centre) will download clinical data to the study office quarterly. Unique database numbers will link these data with the ICON database, allowing record linkage studies with ICNARC. The study office will also snapshot each participating ICU every six months for staff and unit demographics.

### Data protection and PIAG approval

The data collection, storage and access rights will conform to the standards set out in the Data Protection Act 1998. In addition, an application will be made to PIAG (Patient Information Advisory Group) for "Section 60 exemption" class support to provide a basis in law for holding patient identifiable data. This is a requirement in UK law (Health and Social Care Act 2001) for all disease registries. Although the ICON study needs to hold patient identifiable data to allow checking with the ONS, any second parties wishing to use the data will only have access to anonymised information.

### Data Handling

The data from ICNARC databases, the ONS returns, the SF-36, EQ-5D, HADS and PCL-C responses will be stored in a series of linked databases. The data imported as electronic files (ICU databases and ONS records) will undergo filtering, validity and range checks before being included. Data from the paper returns will be scanned and error checked. The data will be held in off-line storage with two-tier backup.

Participating units will receive electronic mailings from the study office giving the number of patients that are currently being followed-up and the average questionnaire results of those patients who have consented to take part in the study.

### Planned analyses

Using ICU admission as the event defining the start of follow-up, Kaplan-Meier curves will be constructed to illustrate survival. Age and sex matched control data will be requested from ONS in a 1:1 ratio with the patients in the study, and a control group Kaplan-Meier curve calculated from these data. A log-rank test will be used to determine the statistical significance of any differences noted.

It is almost certain that the ICU survivors will show a decreased longevity compared with the general population, so this initial testing will only be used to validate the study by showing similar results to previous investigations. However, as this is a non-randomised study, there are many potential sources for bias. To minimise the possibility that a difference in survival is due to confounders and not a real effect of the either illness or the ICU care, further covariate analysis will be undertaken. To determine the effects of covariates on survival, the Cox proportional hazards method will be used. The covariates included in the model will be age, sex, APACHE II predicted mortality, socio-economic status (from postcodes), geographical area (4 zones north to south based on hospital location), month of discharge, and 12 broad diagnostic categories based of the organ system involved in the acute illness. These results will be presented as a table showing the relative effects of ICU care and the covariates on survival.

The data on psychological illness (anxiety, depression and PTSD symptomatology), and quality of life will be largely descriptive and will be presented in tabular form.

### Economic issues

Utility analysis will be performed by estimating Quality Adjusted Life Years (QALYs) from the survival and EQ-5D data at 3, 12 and 24 months using standard techniques. A simple average of QALYs with measures of dispersion will be calculated. Assuming the data prove to be normally distributed, standard parametric testing will be used to compare the mean QALYs in ICU survivors with the age and sex matched controls held in other databases.

## Conclusion

The ICON study is a longitudinal study that seeks to provide rigorous data on the long-term outcome from ICU treatment in the UK, with a particular emphasis on psychological outcomes. Strengths of the study include comprehensive measurement of relevant outcomes and a relatively large projected sample size. Results from the ICON study should help inform policy and guide future research into the care and long-term outcomes of ICU patients.

## Abbreviations

CMP: Case Mix Programme; HADS: Hospital Anxiety and Depression Scale; HRQoL: Health-related Quality of Life; ICNARC: Intensive Care National Audit and Research Centre; ICU: Intensive Care Unit; NHS: National Health Service; ONS: Office of National Statistics; PCL-C: PTSD checklist (civilian); PTSD: Posttraumatic Stress Disorder; PIAG: Patient Information Advisory Group; QALYs: Quality-adjusted life years; SF-36: Short-form 36; UK: United Kingdom.

## Competing interests

The authors declare that they have no competing interests.

## Authors' contributions

JAG, VSB and JDY contributed to the writing of the study protocol. The study was initiated by JDY.

## Pre-publication history

The pre-publication history for this paper can be accessed here:


